# Supplementary material for: WebGWAS: a web server for instant GWAS on arbitrary phenotypes
Source: BioData Min. 2026 Apr 3;19:39. doi: 10.1186/s13040-026-00548-y (PMC13170032; doi:10.1186/s13040-026-00548-y)
Supplement: Supplementary file 1 — Supplementary Material 1 [file 13040_2026_548_MOESM1_ESM.pdf]

SUPPLEMENTARY MATERIALS FOR: WEBGWAS: A WEB SERVER FOR INSTANT GWAS ON ARBITRARY PHENOTYPES

Michael Zietz, Undina Gisladottir, Kathleen LaRow Brown, Nicholas P. Tatonetti

1 Supplementary Methods

1.1 Derivation of Indirect GWAS.

Here we provide a step-by-step derivation of the key indirect GWAS equations presented in the Methods.

1.1.1 Coefficient estimate

Consider the standard OLS coefficient for a regression of residualized phenotype $\tilde{\mathbf{y}}$ on residualized genotype $\tilde{\mathbf{g}}$:

$$\hat{\beta} = \frac{\tilde{\mathbf{g}}^\intercal \tilde{\mathbf{y}}}{\tilde{\mathbf{g}}^\intercal \tilde{\mathbf{g}}}$$

If $\mathbf{y} = \mathbf{X}\mathbf{p}$ (i.e., the phenotype is a linear combination of features), then $\tilde{\mathbf{y}} = \tilde{\mathbf{X}}\mathbf{p}$ because the residual projection is a linear operator ($\mathbf{P}(\mathbf{X}\mathbf{p}) = (\mathbf{P}\mathbf{X})\mathbf{p} = \tilde{\mathbf{X}}\mathbf{p}$). Substituting:

$$\hat{\beta} = \frac{\tilde{\mathbf{g}}^\intercal \tilde{\mathbf{X}}\mathbf{p}}{\tilde{\mathbf{g}}^\intercal \tilde{\mathbf{g}}} = \sum_i p_i \frac{\tilde{\mathbf{g}}^\intercal \tilde{\mathbf{x}}_i}{\tilde{\mathbf{g}}^\intercal \tilde{\mathbf{g}}} = \sum_i p_i b_i = \mathbf{b}^\intercal \mathbf{p}$$

where $b_i = \tilde{\mathbf{g}}^\intercal \tilde{\mathbf{x}}_i / \tilde{\mathbf{g}}^\intercal \tilde{\mathbf{g}}$ is the OLS coefficient from regressing feature i on the genotype.

1.1.2 Standard error estimate

The standard error of $\hat{\beta}$ for a univariate OLS regression is:

$$\mathrm{SE}(\hat{\beta})^2 = \frac{1}{d}\left(\frac{\mathrm{Var}(\tilde{y})}{\mathrm{Var}(\tilde{g})} - \hat{\beta}^2\right)$$

where $d = N - C - 1$ is the degrees of freedom. To apply this for the combined phenotype, we need $\mathrm{Var}(\tilde{y})$ and $\mathrm{Var}(\tilde{g})$. Since $\tilde{\mathbf{y}} = \tilde{\mathbf{X}}\mathbf{p}$:

$$\mathrm{Var}(\tilde{y}) = \mathrm{Var}(\tilde{\mathbf{X}}\mathbf{p}) = \mathbf{p}^\intercal \mathrm{Cov}(\tilde{\mathbf{X}})\mathbf{p} = \mathbf{p}^\intercal \mathbf{C}\mathbf{p}$$

where $\mathbf{C} = \mathrm{Cov}(\tilde{\mathbf{X}})$ is the covariate-adjusted (partial) phenotypic covariance matrix.

1.1.3 Estimating genotype variance

$\mathrm{Var}(\tilde{g})$ is the same for all feature phenotypes (the genotype is shared). Rearranging the standard error formula for each feature phenotype i:

$$\mathrm{Var}(\tilde{g})_i = \frac{\mathrm{Var}(\tilde{x}_i)}{s_i^2 d + b_i^2}$$

In practice, slight numerical differences across GWAS methods and floating-point arithmetic yield slightly different estimates per phenotype, so we take the mean: $\widehat{\mathrm{Var}(\tilde{g})} = \overline{\widehat{\mathrm{Var}(\tilde{g})}_i}$.

Combining all terms yields the final indirect GWAS equations:

$$\hat{\beta} = \mathbf{b}^\intercal \mathbf{p} \qquad \mathrm{SE}(\hat{\beta}) = \sqrt{\frac{1}{d}\left(\frac{\mathbf{p}^\intercal \mathbf{C} \mathbf{p}}{\widehat{\mathrm{Var}(\tilde{g})}} - (\mathbf{b}^\intercal \mathbf{p})^2\right)}$$

1.2 Translation between datasets

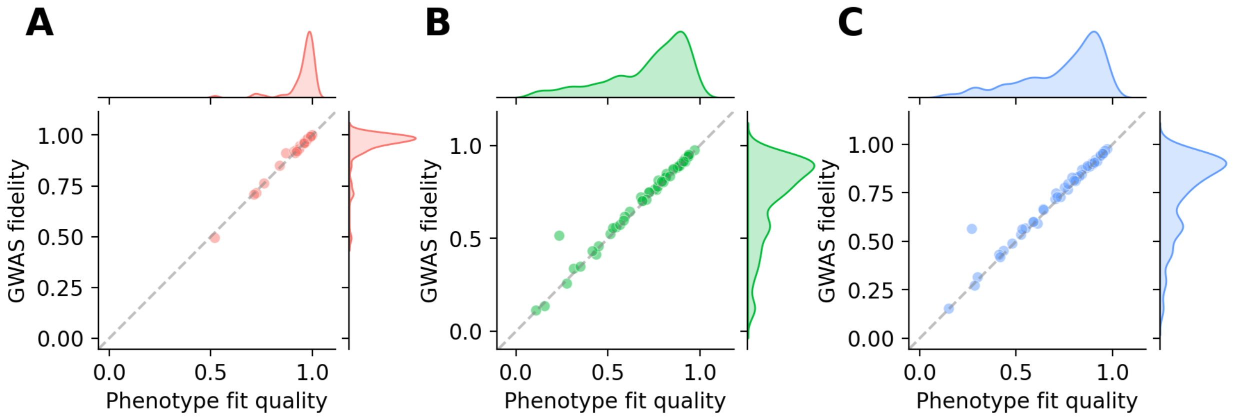

Figure S1: **Indirect GWAS can translate between datasets.** Phecodes were approximated using ICD-10 codes with linear regression for various training and evaluation datasets. Phenotype approximation coefficients were computed in the training dataset. GWAS fidelity and phenotype fit quality were computed in the evaluation dataset. **A:** Phecode approximations from Cedars-Sinai are similar to UK Biobank approximations. Trained in Cedars-Sinai, evaluated against linear approximations from UK Biobank. **B:** Many Phecode approximations from Cedars-Sinai perform well in UK Biobank as well. Trained in Cedars-Sinai, evaluated against true Phecodes in UK Biobank. **C:** For comparison, approximations from the UK Biobank perform similarly. Linear approximation trained in UK Biobank, evaluated against true Phecodes in UK Biobank.

A limitation of indirect GWAS is that it can only operate on phenotypes defined in terms of its input features (i.e. the phenotypes for which a covariance matrix and GWAS summary statistics are available). In examples presented previously in this paper, the features are ICD-10 codes, meaning that indirect GWAS can only approximate phenotypes that are defined in terms of ICD-10 codes. This is limiting, because there are many phenotypes which might be of interest in GWAS but which do not deal with diagnoses and cannot be explicitly defined as such by a researcher. For example, a researcher interested in genetic associations of UK Biobank questionnaire responses could not explicitly define the response in terms of ICD-10 codes.

However, a large set of real human phenotypes, such as measurements and diagnoses, will span a reasonable fraction of the phenotypes of interest for GWAS. This means that a large dataset like this should have a reasonable amount of statistical predictive power for many real human phenotypes. In some cases, that predictive power could be large enough that a user could reasonably approximate their phenotype of interest using features included in WebGWAS, and use that approximation to gather immediate, approximate GWAS summary statistics. In short, for example, a researcher interested in a particular phenotype could train a statistical or machine learning model to approximate the phenotype using diagnoses and measurements as features, and obtain approximate GWAS summary statistics for that phenotype using WebGWAS. This would be particularly valuable if they could train this model in completely separate phenotype data, for example, an entirely different cohort than the UK Biobank.

Whether this is possible depends on several factors. First, the phenotype of interest needs to be approximable using the features that WebGWAS includes. In building WebGWAS, we attempted to include as many features as possible, to maximize this flexibility for users. However, the space of real human phenotypes is vast, and there are undoubtedly many phenotypes which cannot be approximated well using the features WebGWAS includes. Second, the statistical relationships between the phenotype of interest and the features need to be similar between datasets. For example, even if a user can approximate questionnaire responses using ICD-10 codes and measurements in EHR data from a hospital, this can only translate to good performance in WebGWAS if the correlations among features and the questionnaire are

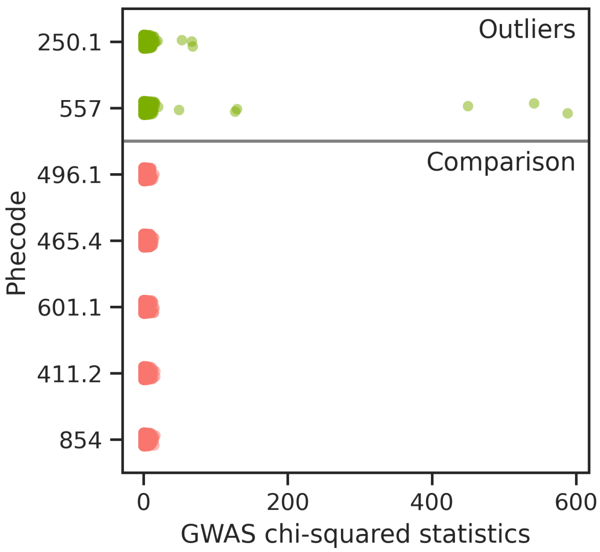

Figure S2: Two outlier phenotypes had moderate phenotype fit quality but exceptional GWAS fidelity, as shown in Figure 3. Upon investigation, the reason for this exceptional GWAS fidelity is that they are outliers in terms of GWAS chi-squared statistics, which leads to inflated variance of chi-squared statistics and inflated Pearson correlation.

similar in the UK Biobank cohort as in the EHR cohort. This is a fundamental limitation of a cross-dataset approach, and it cannot easily be evaluated statistically without individual-level data from both cohorts.

In a simple evaluation, we found that Phecode approximations translate very well between a large EHR cohort and the UK Biobank (Figure S1). As features, we used ICD-10 code diagnosis data from Cedars-Sinai Medical Center. We gathered data for 491,822 patients at Cedars-Sinai, and took all 266 ICD-10 codes that had at least 1000 cases in both the UK Biobank and Cedars-Sinai datasets. We then constructed all non-trivial Phecodes that could be constructed with these ICD-10 codes (N=52). In both datasets, we ran linear regressions to approximate the Phecodes using the ICD-10 codes. Finally, we evaluated both regressions in the UK Biobank to obtain three definitions for each Phecode: one exact, one using a UK Biobank linear approximation, and one using a Cedars-Sinai linear approximation.

1.3 Investigation of outlier performance

In Figure 3, we noticed that two Phecodes showed GWAS fidelity far above their phenotype fit quality. These two Phecodes were 250.1 (Type 1 diabetes) and 557 (Intestinal malabsorption (non-celiac)). We investigated and found that both were among the lowest prevalence Phecodes in this analysis. Upon further investigation, we discovered that these two phenotypes were outliers in terms of their GWAS summary statistics (Figure S2). Both had a very small number of extreme associations, far beyond the distribution of effects observed for other phenotypes. These outlier variants were also well-approximated with our indirect approach (Figure 2), and they therefore inflated the GWAS fidelity by massively increasing the variance of the chi-squared statistics. Overall, this is an artifact that appears because we used a limited number of genetic variants for this analysis. Our target outcome variable was GWAS fidelity, not genome-wide statistical significance, so we reduced the number of variants that we considered in order to improve computation times. These outliers are in the direction of better fit, not worse, so we believe that they represent a limitation with our evaluation metric (GWAS fidelity) rather than an issue with the method.
